# Evolution of the educational system and micro-psychological mechanisms: a comparative study of academic motivation and engagement in Shanghai, China and Kazakhstan

**DOI:** 10.3389/fpsyg.2026.2005404

**Published:** 2026-10-28

**Authors:** Zhan Zhou, Aigul Alchimbayeva, Xiaogang Jin

**Affiliations:** 1 Faculty of Philosophy and Political Science, Al-Farabi Kazakh National University, Almaty, Kazakhstan; 2 Faculty of Arts, Huanggang Normal University, Huanggang, China

**Keywords:** cross-cultural psychology, educational environment perception, Intrinsic Motivation, Learning Engagement, measurement invariance, Self-Determination Theory

## Abstract

**Introduction:**

As noted in successive OECD assessment reports, global education systems are shifting from standardized assessments toward prioritizing students’ psychological wellbeing and autonomous agency. However, cross-cultural quantitative evidence regarding how these macro-policy transformations relate to students’ micro-psychological perceptions and academic behaviors remains limited.

**Methods:**

Utilizing a cross-sectional survey, this study analyzed data from 1,309 university students in Shanghai, China, and Kazakhstan. Multi-Group Confirmatory Factor Analysis and Structural Equation Modeling (SEM) were employed to examine the structural associations and measurement invariance among Perceived Educational Transformation (PET), Intrinsic Motivation (IM), and Learning Engagement (LE).

**Results:**

The three-factor model demonstrated strict measurement invariance across both samples. SEM findings indicated that IM partially mediated a significant indirect associative mechanism the positive structural relationship between PET and LE (indirect effect = 0.098, 95% CI [0.076, 0.122]). This statistical mediating pathway was consistent across both cohorts, showing only trivial inter-group differences (all parameter Δ*p* > 0.05). Baseline comparisons revealed the Shanghai sample reported higher PET, yet IM and LE levels exhibited no significant quantitative differences between the two groups (Cohen’s *d* < 0.10).

**Discussion:**

Higher perceptions of educational transformation do not linearly correspond to enhanced motivation, potentially constrained by unobserved systemic evaluation pressures. The findings suggest that autonomy-supportive educational environments are consistently associated with higher Intrinsic Motivation and academic engagement, regardless of differentiated macro-educational contexts. However, due to the cross-sectional design, these findings represent structural associations rather than directional causality.

## 1 Introduction

### 1.1 Global educational assessment and the shift in paradigm evolution

Over the past two decades, the field of international educational assessment has continuously undergone a gradual paradigm reconstruction. In this process, the Programme for International Student Assessment (PISA) led by the OECD is not only a macro-recorder of this change but also, to a considerable extent, anchors the agenda direction of educational reforms in various countries (Seitzer et al., 2021).

The 2012 and 2015 PISA reports (OECD, 2013, 2017) revealed an internal tension universally existing in high-achievement education systems: students in some countries and regions perform excellently in standardized academic tests, yet simultaneously exhibit lower internal driving forces for learning, high-pressure academic anxiety, and a sense of alienation from school life. This structural contradiction reflects a latent value deviation at the systemic level—when the operational focus of the educational system overly concentrates on quantifiable hard performance outputs, the endogenous psychological demands and adaptive states of the learning individuals within the system often slip into peripheral variables.

Subsequently, the 2018 PISA report systematically expanded its inspection perspective to the dimensions of students’ campus life experiences and psychological wellbeing (OECD, 2019); and by 2022, against the backdrop of reshaping the global educational ecology, the assessment further listed students’ autonomous agency and systemic adaptability, accumulated in the previously complex system, as core concerns (OECD, 2023). From this diachronic context, the evolution of educational systems across the global dimension has clearly shown an essential transition from a pure “standardized cognitive output orientation” to an “equal emphasis on the adaptation process and individual psychological foundation.”

This macro-concept evolution, originating in the primary and secondary education foundation measurement field, is by no means an isolated cross-sectional phenomenon but deeply overlaps with the higher education stage in line with the talent cultivation echelon. As the final stage connecting campus and society, the teaching ecology of universities is currently also facing the inevitable requirement of paradigm adjustment—gradually shifting from an emphasis on general standardized assessments and competitive diversion to a focus on inquiry autonomy, complex context tolerance, and internal motivation for knowledge. Crucially, the current generation of university students is precisely the group that has grown up completely accompanying this global educational reform wave lasting for over a decade. As the terminal outcome of these foundational education shifts, when they enter higher learning institutions and directly face more exploratory micro-teaching contexts, the adaptive posture reflected at their psychological system level has already constituted an ideal empirical entry point for testing the long-term relevance of macro-educational reform concepts.

However, although the above macro-level evolutionary trends have already been laid out in the top-level higher education policy texts of many countries, to what extent does this systemic value reset substantively penetrate the daily cognition of the educated? It remains methodologically challenging to directly equate decadal policy shifts with individual psychological states. Such core questions currently still lack sufficient and detailed empirical references, which also constitutes the core basis for the in-depth exploration of this study.

### 1.2 Research gap at the micro-psychological level

Between the alternation of macro-educational policies and the final observable terminal academic behaviors of students, there has long been a longitudinally cut intermediate zone that has not been fully measured so far—namely, the subjective cognitive reconstruction of students regarding specific environmental reform indicators and its reflection path in the deep psychological motivation structure. Currently, theoretical works based on the perspective of educational sociology comparing policy texts are relatively abundant in academia; however, regarding the extent to which top-down value transformation signals are routinely accepted by learners and leave statistically verifiable associated imprints in their psychological dynamic systems and actual engagement (Bureau et al., 2022; Ryan and Deci, 2020), there is still an empirical testing gap—especially when extended horizontally to cross-cultural empirical measurements, this gap is particularly significant.

Based on the above background, this study converges grandiose abstract questions into quantitative testing at the operational dimension, innovatively operationalizing them into the structural association of three core constructs: the tested group’s Perceived Educational Transformation (PET), Intrinsic Motivation (IM) arousal, and daily Learning Engagement (LE) level. It must be emphasized that what PET captures is not a stereotyped objective measurement of the promulgation of policy provisions, but rather the students’ subjective qualitative judgment of whether the environment is “autonomy-supportive and trial-and-error inclusive” when they personally experience micro-teaching interactions.

To avoid conflating macro-level policy enactment with micro-level psychological realities, this study positions PET explicitly as a micro-level perceptual proxy rather than a direct metric of macro-policy evolution. Maintaining this distinction is essential, as examining students’ localized, proximal learning environments remains a scientifically rigorous step in understanding contemporary motivation and engagement (Jiang and Wang, 2026; Yang, J. et al., 2025). Within this micro-framework, IM measurement excludes external utilitarian fetters and accurately reflects students’ original tendency to maintain academic attention purely based on flow experience and zeal for knowledge; LE three-dimensionally integrates cognitive concentration, overt behavior, and underlying emotion, thereby anchoring the individual’s comprehensive immersion scale in real scenarios. In summary, the indirect structural association presented among these three major constructs—especially examining whether IM builds a substantive structural bridge in the relationship where PET relates to LE, and whether this associative structure reveals differential divergences under the moderation of macro-cultural contexts (Assefa et al., 2025)—naturally extends as the core empirical axis anchored by this study.

### 1.3 Choice of comparative contexts: Shanghai, China, and Kazakhstan

After establishing the aforementioned micro-psychological mechanism model, the premise for testing its cross-contextual stability lies in selecting comparative groups that can represent heterogeneous evolutionary directions of macro-educational systems. This study selects the Shanghai sample in China and Kazakhstan as the macro-comparative framework. Given the regional development differences under China’s massive educational volume, this study specifically selects Shanghai, which has long served as a national comprehensive educational reform experimental zone and deeply participated in PISA assessments, as the fixed group sampling area to represent a highly intensive regional reform context within China.

The institutional evolution in the Shanghai sample area presents the characteristics of intra-system gradual improvement. Its educational environment simultaneously bears the high-intensity performance screening mechanism unique to East Asia, as well as the educational reform attempts continuously promoted at the official level in recent years, which are oriented toward core competencies, de-emphasizing test-taking, and diversifying evaluation (Deng et al., 2024; Luo, 2023; Wei et al., 2025). Against this background, the student population is situated in an educational environment where competitive pressure and institutional improvement intertwine.

In contrast, the educational evolution of Kazakhstan follows the macro-logic of transitional reconstruction (Bridges, 2014). Since the dissolution of the Soviet Union, the country has continuously promoted systemic reconstruction centered on international integration (e.g., full inclusion in the European Bologna Process; Duman, 2024; Narbaev et al., 2025; Silova and Steiner-Khamsi, 2008). This reform is accompanied by large-scale parallel resets of curriculum standards, credit recognition, and educational language policies. Unlike the endogenous gradual improvement continuously promoted by the Chinese side, Kazakhstan’s reforms inherently reflect more significant characteristics of institutional deconstruction and exogenous replacement (Kuzhabekova, 2025; Yakavets et al., 2023).

The fundamental substantive differences in the historical reform logic of the two contexts mentioned above (gradual improvement vs. transitional integration) provide a rigorous macro-environmental variable foundation for this study to place the two groups of subjects under a multi-group comparative framework and quantitatively examine the performance similarities and differences of the same micro-psychological association structure in different institutional evolution models.

### 1.4 Research questions

Based on the aforementioned research gaps and macro-contextual design, this study intends to conduct quantitative testing on the following three major practical questions:

*RQ1*: Do university students in Shanghai and Kazakhstan exhibit statistically significant inter-group differences at the baseline levels of the core observed variables: Perceived Educational Transformation (PET), Intrinsic Motivation (IM), and Learning Engagement (LE)?

*RQ2*: Within the combined full sample range, does the positive measurement relationship between Perceived Educational Transformation (PET) and Learning Engagement (LE) present an indirect link systematically connected by Intrinsic Motivation (IM)?

*RQ3*: Does the overall architecture and various path strengths of this “PET—IM—LE” central association system maintain cross-cultural equivalence within these two specific institutional samples of Shanghai and Kazakhstan, or do they show evidence of divergence moderated by macro-educational circumstances at the statistical level?

## 2 Theoretical framework and research hypotheses

The continuous evolution of the focus of global educational assessments provides a macro-background for examining students’ Perceived Educational Transformation, Intrinsic Motivation, and Learning Engagement from a micro-psychological level, as specifically shown in Figure 1.

**Figure 1:**
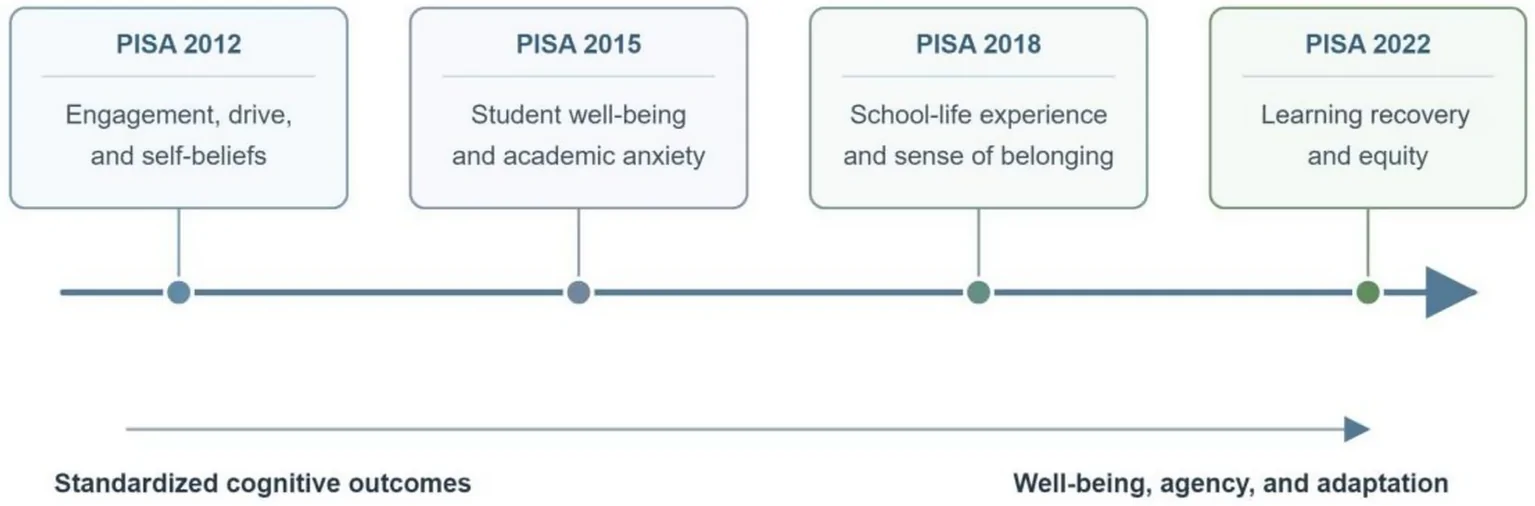
Evolution of the focus of global educational assessments since 2012. Compiled based on successive OECD PISA reports.

Under this macro-shift, Self-Determination Theory (SDT) provides an appropriate theoretical framework for explaining how the perception of the educational environment is associated with students’ Intrinsic Motivation and Learning Engagement.

### 2.1 Self-Determination Theory (SDT) and educational system adaptation

The theoretical deduction of this study is rooted in Self-Determination Theory (SDT). This theoretical framework (Ryan and Deci, 2000; Deci and Ryan, 2013) has accumulated a large amount of empirical support within the educational domain (King and McInerney, 2014; Lo, 2024). Its core proposition is that the survival quality of the internal motivation system of learning individuals depends to a considerable extent on whether the external environment can fulfill their three basic psychological needs—autonomy, competence, and relatedness.

Juxtaposing SDT within the coordinates of macro-educational system evolution, its theoretical implication is mainly reflected here: the alternation of the overall educational orientation, by shaping the satisfaction baseline of students’ basic psychological needs, is expected to present a specific co-variation association with students’ Intrinsic Motivation state. This observational model does not pre-assume a linear physical correspondence between macro-policy texts and individual micro-psychology, but creatively places students’ subjective realization of environmental transformation—namely PET—at the forefront of the perceptual interface. Through this lens, Perceived Educational Transformation (PET) translates abstract systemic shifts into measurable proximal experiences, operationalized specifically as students’ daily perceptions of autonomy-supportive climates, tolerance for exploratory errors, and competency-oriented teaching in their immediate learning environments. Anchored in these daily classroom realities, SDT provides a solid theoretical backing for sorting out the structural coupling of the three core constructs of PET, IM, and LE; at the same time, it also reserves a reasonable interpretive space for exploring the moderating heterogeneity of cross-cultural ecologies—the supply threshold survival differences and support structures of different cultural systems for students’ sense of autonomy and competence (Yang, Y. et al., 2025). This means that even if Shanghai and Kazakhstani students report equal levels of PET, the IM active parameters mapped deeply in their psychological structures may also reveal different tendencies at the quantitative statistical level.

### 2.2 Macro-institutional background and baseline difference hypotheses

The university sample areas in Shanghai and Kazakhstan are long embedded in educational evolutionary logics of vastly different natures. The Shanghai system possesses highly intensive gradual improvement characteristics; discussions on educational reform discourses around “breaking the obsession with scores” and “encouraging inquiry and innovation” at the official policy level show extremely high systemic heat. In contrast, Kazakhstan’s evolutionary process relies more on holistic de-traditionalized Western integration and reconstruction. Combining SDT principles, an individual’s observational acuity to environmental signals is usually positively correlated with the release frequency of external information sources. Situated in the Shanghai region, activated with high frequency by both educational reform volume and policy advocacy, it is expected that the test group’s ability to capture these contextual autonomy-supportive and competency-based representations in micro-classrooms will be relatively stronger.

However, looking back at the baseline dimensions of Intrinsic Motivation (IM) and practical Learning Engagement (LE), the checking and balancing effect of the macro-system may intertwine into a more complex state of tension. Although the higher teaching ecologies of both contexts are undergoing deconstruction, in the higher-level system, individuals in both regions also face extremely oppressive social mobility and fierce employment and further education screening mechanisms (Li, 2012; Zhang et al., 2023). Because these overarching systemic pressures were not explicitly measured, deducing a straightforward, unidirectional inter-group difference for motivational and behavioral outcomes remains theoretically cautious. Accordingly, this study proposes the following array of difference hypotheses regarding baseline indicators:

*H1a*: Based on the higher localized density of pedagogical reform practices, the baseline mean of Perceived Educational Transformation (PET) of the test group in Shanghai is estimated to significantly exceed that of the Kazakhstan group.

*H1b*: The Shanghai and Kazakhstan samples are expected to have no obvious inter-group differences at the baseline levels of Intrinsic Motivation and Learning Engagement, or only present trivial differences of limited practical significance.

### 2.3 Indirect structural association of Intrinsic Motivation

Inheriting the dynamic generation mechanism concept of SDT, an individual’s environmental perception of autonomy-supportive teaching changes does not directly and losslessly translate into mechanical behavioral increments. The theory advocates that when the environment provides signals of inclusion and autonomy support, the primary reflection of this cognitive state should be the simultaneous symbiosis of internalized thirst for knowledge and exploratory interest (i.e., the positive coupling of PET and IM); and only when an individual’s Intrinsic Motivation is fully aroused can it substantively covary with deep academic engagement in cognitive participation, overt actions, and emotional concentration. This preliminary hypothesis strips away the direct induction of the environment on behavior and explicitly identifies the statistically indirect association role of IM in this triangular structural framework (Bing, 2026). Accordingly, the associated hypotheses are derived:

*H2*: In the overall test structure, there is a positive statistical association between Perceived Educational Transformation (PET) and final Learning Engagement (LE).

*H3*: Intrinsic Motivation (IM) exerts a systematic positive indirect associative link in the structural relationship where PET links to LE, meaning that a statistically supported proportion of the variance in environmental perception (PET) is statistically linked to engagement through the accompanying Intrinsic Motivation (IM).

### 2.4 Moderating hypothesis of transnational macro contexts

Based on the cross-sectional difference characteristics of the educational systems in the Shanghai sample and Kazakhstan, this study estimates that their divergence is not merely limited to the original dropping of front-end isolated parameters (such as H1) but is more likely to dive into the deep valley of the structural mechanism framework, such that a specific association path presents inter-group differences in strength. While SDT assumes general cross-cultural applicability, the vastly different baseline conditions of the two systems—gradual internal reform intertwined with rigid competitive screening in Shanghai versus rapid Western integration in Kazakhstan—might theoretically foster differential sensitivities to autonomy-supportive signals. This provides a supporting foothold in the statistical dimension for exploratory testing, expecting that the Shanghai cross-section may show significantly stronger characteristics than the Kazakh region regarding the coefficient loading of the PET-IM association.

*H4*: The national-level external educational system variable (Shanghai sample vs. Kazakhstan region) exhibits a moderating role within the overall structural model—especially on the front-end path (between PET → IM). That is, the Shanghai sample is expected to show stronger positive association characteristics in this path.

Based on the above theoretical analysis, this study constructs a theoretical model of Perceived Educational Transformation, Intrinsic Motivation, and Learning Engagement, as specifically shown in Figure 2.

**Figure 2:**
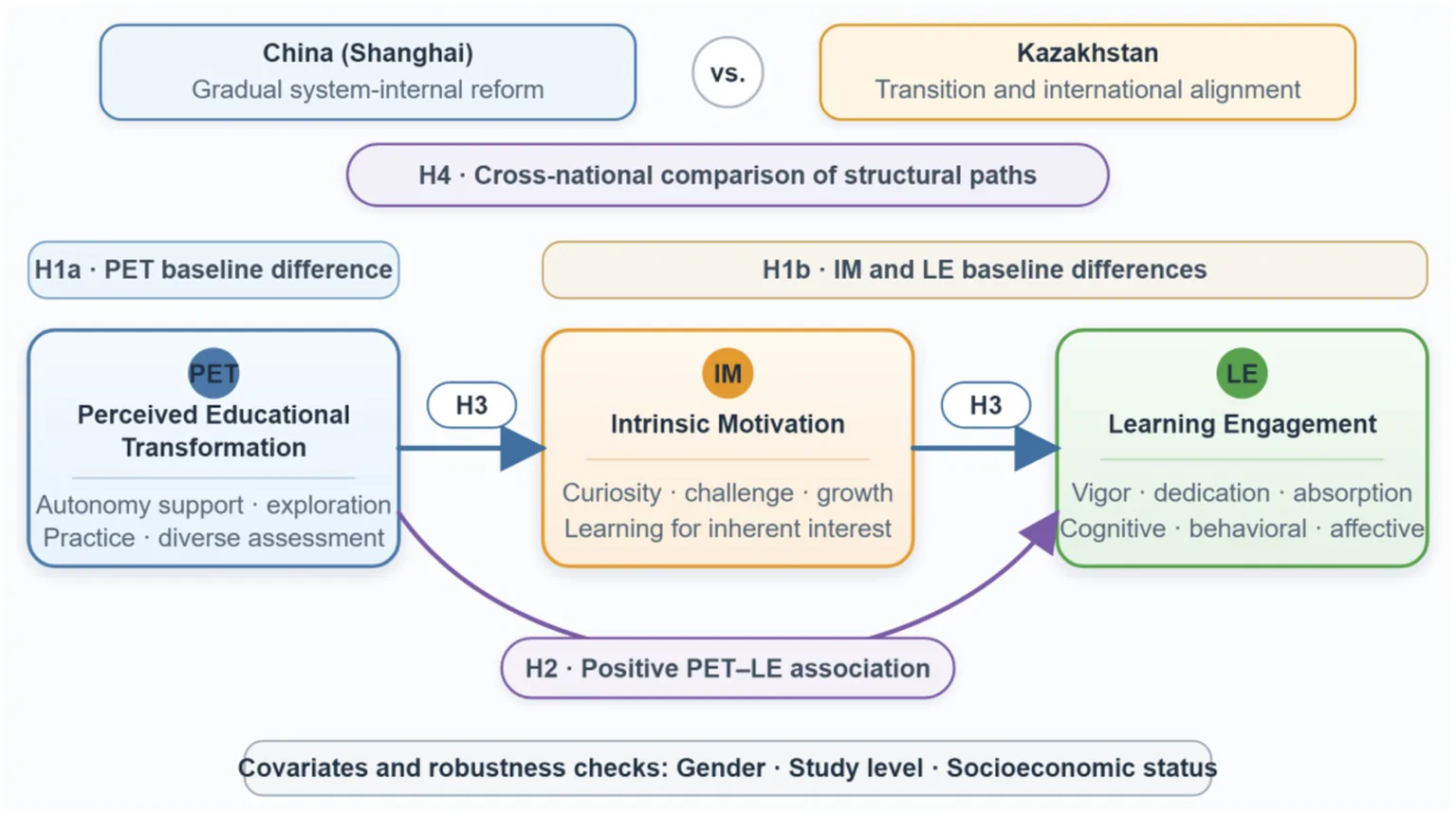
Theoretical structural association model of Perceived Educational Transformation, Intrinsic Motivation, and Learning Engagement. PET = Perceived Educational Transformation; IM = Intrinsic Motivation; LE = Learning Engagement. Given the cross-sectional nature of this study, single-headed arrows strictly denote hypothesized statistical structural associations and indirect pathways grounded in SDT, rather than chronological causal relationships.

## 3 Research methods

### 3.1 Data sources and participants

This study adopted a cross-sectional questionnaire survey design. To closely align with the theoretical focus on distinct macro-educational transitions, a purposive sampling strategy was employed. Data were collected simultaneously from university networks in Shanghai, China—a representative hub for intensive domestic pedagogical reform—and cooperative universities in Kazakhstan, which serve as active nodes for international higher education integration (e.g., the Bologna Process). To avoid sample homogenization bias stemming from single-faculty clustering, the research team systematically covered core professional groups such as humanities, science and engineering, and medicine through the teaching networks of cooperative universities in both places. The target population was limited to enrolled university students aged 18 and above (including all training stages from undergraduate to doctoral). To ensure the equivalence of cross-cultural comparison, the questionnaire used the original English version as a benchmark, strictly underwent a bilingual back-translation procedure, and was presented in Chinese (for the Shanghai sample) and Russian (for the Kazakhstan sample), consistent with the primary mediums of instruction within these specific institutional contexts. The complete multilingual comparative questionnaire and scoring codebook are detailed in the Supplementary materials. All subjects voluntarily completed the informed consent confirmation before formally entering the questions; the questionnaire was strictly anonymous, and the data were solely used for academic purposes.

Data cleaning followed strict operational guidelines: sequentially eliminating records that failed the informed consent and age/identity screening questions, erroneous values on the attention check question (Question 17), and invalid questionnaires with regular answering patterns (e.g., straight-line answering by selecting the same option). At the level of outlier detection, the standardized *Z*-scores for each scale’s score were uniformly calculated, and extreme value records falling outside the range of ±3 standard deviations were excluded. Furthermore, given that the online survey system had embedded a mandatory response mechanism for core scales, there were no missing values at the item level in the raw data, thereby completely avoiding statistical biases introduced by multiple imputation or listwise deletion.

After the aforementioned data screening procedures, the Shanghai sample converged from the initial 720 to 698 copies, and the Kazakhstan sample converged from 630 to 611 copies. The final total valid sample size for the two countries was 1,309 (the effective rate exceeded 96% for both). Regarding the demographic composition of the sample, both groups were predominantly undergraduate students, covering all grade levels from freshman to doctoral; specific distributions of basic information such as gender composition, major categories, and family economic status are detailed in Table 1.

**Table 1:** Basic demographic characteristics of the sample (*N* = 1,309).

Variable	Category	Shanghai *n* (%)	Kazakhstan *n* (%)
Gender	Male	302 (43.3%)	286 (46.8%)
	Female	380 (54.4%)	316 (51.7%)
	Other/Undisclosed	16 (2.3%)	9 (1.5%)
Study Status	Undergraduate	522 (74.8%)	471 (77.1%)
	Master’s student	137 (19.6%)	97 (15.9%)
	Doctoral student	39 (5.6%)	43 (7.0%)
Age	M (SD)	21.4 (3.2)	21.4 (3.4)
	Range	18–34	18–34
Major Category	Humanities and Social Sciences	216 (30.9%)	189 (30.9%)
	Science and Engineering	271 (38.8%)	237 (38.8%)
	Medicine and Health	81 (11.6%)	56 (9.2%)
	Education	64 (9.2%)	61 (10.0%)
	Arts and Sports	26 (3.7%)	34 (5.6%)
	Other	40 (5.7%)	34 (5.6%)
Family Economic Status	Significantly below average	34 (4.9%)	29 (4.7%)
	Slightly below average	106 (15.2%)	87 (14.2%)
	Average level	357 (51.1%)	299 (48.9%)
	Slightly above average	137 (19.6%)	129 (21.1%)
	Significantly above average	64 (9.2%)	67 (11.0%)
Self-rated Academic Performance	Very unsatisfactory/Not quite satisfactory	65 (9.3%)	59 (9.7%)
	Average	285 (40.8%)	245 (40.1%)
	Good/Very good	348 (49.9%)	307 (50.2%)

### 3.2 Measurement tools

The core constructs involved in this study were all measured using validated self-report scales. Conceptually, this measurement system constitutes a complete chain from environmental cognition to psychological arousal and then to behavioral output. All items adopted a 5-point Likert scoring format (1 = *completely disagree/never*, 5 = *completely agree/always*).

#### 3.2.1 Perceived Educational Transformation (PET) scale

To measure this construct, the present study adapted 8 highly representative items from established learning environment inventories. Rather than directly adopting macro-policy measurement items, the scale operationalizes educational transformation as students’ subjective feelings in the micro-teaching environment, drawing its conceptual framework and initial item structures primarily from constructivist learning environment research (Taylor et al., 1997) and classical autonomy-supportive climate measures (Williams and Deci, 1996), with localized textual modifications to reflect the ongoing systemic transition away from traditional test-oriented paradigms.

The adapted scale specifically captures three key orientations: liberation of cognition and thinking (e.g., “teachers encourage expressing opinions different from standard answers,” tolerating exploratory mistakes; Items 14.1, 14.4, 14.7, 14.8), practice and competency orientation (e.g., using knowledge to solve real-world problems, emphasizing communication; Items 14.2, 14.3), and the evolution of the evaluation system. In the analytical model, rather than treating these as separate subscales, they are treated as manifest indicators aggregated into a unidimensional composite latent variable to reflect a holistic perception of systemic pedagogical shifts. It is worth noting that items 14.5 and 14.6 (measuring whether course evaluation relies solely on rote memorization and exam results), as positive statements of traditional test-oriented approaches, were strictly reverse-scored to align with the conceptual direction of the scale. After conversion, a higher total score on this scale implies that students perceive the educational environment as leaning more toward supporting autonomy and capability development.

#### 3.2.2 Intrinsic Motivation (IM) scale

Students’ deep academic motivation was measured using an 8-item scale. This scale follows the development logic of the Intrinsic Motivation dimension in the Academic Motivation Scale (AMS) by Vallerand et al. (1992), taking Self-Determination Theory (SDT; Ryan and Deci, 2000) as the core theoretical framework. In terms of item layout, this scale strips away external instrumental incentives and highly focuses on three internal experiences induced by the learning activity itself: satisfaction of the thirst for knowledge (e.g., “enjoying the pleasure of discovering new knowledge”), achievement experience after cognitive challenges (e.g., “sense of achievement from solving difficult problems”), and the pure sense of internal growth acquisition. All items are positively scored; a higher score indicates a more active state of Intrinsic Motivation arousal.

#### 3.2.3 Learning Engagement (LE) scale

The measurement of this construct adopted the highly recognizable classic tool adapted from Schaufeli et al. (2002)—the 9-item version of the Utrecht Work Engagement Scale for Students (UWES-S). This scale three-dimensionally reconstructs the three core pillars of Learning Engagement: vigor (assessing physical and mental readiness and resilience, e.g., “feeling energetic”), dedication (assessing emotional identification and enthusiasm for the meaning of learning), and absorption (measuring an immersion experience close to “flow,” e.g., “time flies when I am studying hard”). The effective aggregation of these three dimensions provides a solid terminal indicator for examining the co-variation relationship between Intrinsic Motivation and high-quality behavioral engagement at the statistical association level. All items are positively scored.

### 3.3 Data analysis strategy

The data analysis followed a progressive logic from measurement to structure, and from description to inference, unfolding sequentially across three levels.

First level: Data quality assessment. Before entering formal analysis, Harman’s single-factor test, complemented by a Common Latent Factor (CLF) analysis within the SEM framework, was used to evaluate the potential interference of Common Method Bias (CMB) (Hair et al., 2010); the distributional characteristics of each variable were verified through skewness and kurtosis indicators and the *K*-S test, verifying the assumptions for subsequent parameter estimation.

Second level: Measurement model testing. Given that the Perceived Educational Transformation (PET) scale was synthesized and adapted from multiple established sources to fit a novel cross-national sample comparison, the study first utilized Confirmatory Factor Analysis (CFA) to verify the measurement models’ construct validity and structural configurations, comprising the three latent variables of PET, IM, and LE. Fit indices such as *χ*^2^/*df*, CFI, TLI, RMSEA, and SRMR were reported, and reliability and validity were assessed in combination with Composite Reliability (CR), Average Variance Extracted (AVE), and correlation coefficients among constructs. Based on this, Multi-Group Confirmatory Factor Analysis (MGCFA) was employed to sequentially test the configural invariance, loading invariance, intercept invariance, and strict invariance between the Shanghai and Kazakhstan samples. Referring to the recommendations of Chen (2007) and Cheung and Rensvold (2002), ∣ΔCFI∣ ≤ 0.010 and ΔRMSEA ≤ 0.015 were used as the primary judgment criteria, and measurement invariance was comprehensively judged in combination with SRMR changes and chi-square difference tests.

Third level: Structural hypothesis testing. Regarding RQ1 (H1), independent samples *t*-tests utilizing observed composite scores were used to compare the inter-group differences in the mean levels of the three variables between the two sample cohorts, reporting Cohen’s *d* effect sizes. Regarding RQ2 (H2, H3), Pearson correlation analysis provided the bivariate association matrix, followed by Structural Equation Modeling combined with the Bootstrap resampling method (5,000 resamples, with effect significance evaluated via 95% bias-corrected confidence intervals excluding zero) to test the indirect structural association of PET → IM → LE. Regarding RQ3 (H4), based on establishing measurement invariance, Multi-Group Structural Equation Modeling (MGSEM) was used to, respectively, estimate the structural paths in the Shanghai and Kazakhstan samples, and the inter-group coefficient difference test was used to investigate whether key paths possessed significant inter-group differences. Demographic heterogeneity testing served as a supplementary analysis explored through sub-group comparisons of gender and educational level via independent samples *t*-tests. All analyses were completed in the Python (semopy, scipy) environment.

## 4 Research results

### 4.1 Common Method Bias and measurement model testing

#### 4.1.1 Evaluation of Common Method Bias (CMB)

Given that the core constructs of this study all originated from subjects’ self-reports at the same time point, this study first applied Harman’s single-factor test to conduct an unrotated principal component analysis on all 25 scale items (Podsakoff et al., 2003) to evaluate the potential interference of CMB. The results extracted multiple factors with eigenvalues greater than 1, where the variance explained by the first principal factor was 25.33%, far below the critical threshold of 50%. To further ensure robustness beyond exploratory techniques, a Common Latent Factor (CLF) analysis was subsequently conducted within the CFA framework. The inclusion of an unmeasured latent method factor did not result in any substantial changes to the standardized factor loadings (all Δ*λ* < 0.200). This dual-validation approach indicates that there is no single factor dominating the sample variance distribution in the data, and the systematic co-variation overlap of the measurements is within an acceptable range. The distribution indicators of the composite scores of the three constructs were all within the acceptable range (absolute value of skewness < 0.25, absolute value of kurtosis < 0.35), approximating a normal distribution and satisfying the basic assumptions of maximum likelihood estimation.

#### 4.1.2 Confirmatory Factor Analysis (CFA), reliability, and validity

Confirmatory Factor Analysis (CFA) with Maximum Likelihood Estimation (MLE) was utilized to verify the measurement models’ construct validity. The measurement model demonstrated excellent fit to the data (*χ*^2^(272) = 304.36; *χ*^2^/*df* = 1.12; CFI = 0.996, TLI = 0.995; RMSEA = 0.010).

At the level of construct reliability and validity, as shown in Table 2, the Cronbach’s α coefficients of the three scales all exceeded 0.79; the Composite Reliability (CR) ranged from 0.847 to 0.867, showing good internal consistency. Although the Average Variance Extracted (AVE) values were marginally below 0.50, they did not shake the basic requirement of CR > 0.70. To establish a more robust evaluation of discriminant validity alongside the criterion proposed by Fornell and Larcker (1981), the Heterotrait-Monotrait (HTMT) ratio of correlations (Henseler et al., 2015) was additionally assessed. The calculated HTMT values among PET, IM, and LE ranged from 0.412 to 0.528, operating well below the strictly recommended 0.85 benchmark. Based on these combined metrics, the discriminant validity of the constructs was robustly confirmed. Furthermore, all standardized factor loadings ranged from 0.613 to 0.675, retaining all original items.

**Table 2:** Scale reliability coefficients and composite reliability (CR).

Scale	No. of items	Full sample *α*	Shanghai *α*	Kazakhstan *α*	CR	AVE
PET (Perceived Educational Transformation)	8	0.793	0.783	0.800	0.847	0.409
IM (Intrinsic Motivation)	8	0.814	0.829	0.794	0.860	0.435
LE (Learning Engagement)	9	0.827	0.832	0.820	0.867	0.420

CR and AVE were calculated based on CFA standardized factor loadings.

#### 4.1.3 Multi-group measurement invariance testing

To ensure that the means and structural paths between the Shanghai and Kazakhstan samples are comparable, this study further adopted Multi-Group Confirmatory Factor Analysis (MGCFA) to sequentially test the configural invariance, loading invariance, intercept invariance, and strict invariance of the three-factor measurement model of PET, IM, and LE. Using ∣ΔCFI∣ ≤ 0.010 and ∣ΔRMSEA∣ ≤ 0.015 as the primary judgment criteria, comprehensive judgments were made in combination with SRMR changes and chi-square difference tests. The results are shown in Table 3.

**Table 3:** Multi-group measurement invariance testing for Shanghai and Kazakhstan samples.

Model	*χ* ^2^	*df*	CFI	TLI	RMSEA	SRMR	Δ*χ*^2^	Δ*df*	*p*Δ*χ*^2^	ΔCFI	ΔRMSEA	ΔSRMR
Configural Invariant Model	568.307	544	0.997	0.997	0.006	0.030	–	–	–	–	–	–
Loading Invariant Model	598.692	566	0.996	0.995	0.007	0.032	30.385	22	0.109	−0.001	0.001	0.002
Intercept Invariant Model	617.732	588	0.996	0.996	0.006	0.032	19.040	22	0.643	<0.001	<0.001	<0.001
Strict Invariant Model	633.334	613	0.997	0.997	0.005	0.033	15.602	25	0.926	0.001	−0.001	0.001

The loading invariant model is compared with the configural invariant model; the intercept invariant model is compared with the loading invariant model; the strict invariant model is compared with the intercept invariant model. ΔCFI, ΔRMSEA, and ΔSRMR are calculated by subtracting the previous level model from the current model.

The overall fit of the configural invariant model reached a good level, indicating that PET, IM, and LE have a consistent basic factor structure in both the Shanghai and Kazakhstan samples. After sequentially imposing equality constraints on loadings, intercepts, and residual variances based on this, the fit indices of the models at each level did not show obvious deterioration: the changes in CFI, RMSEA, and SRMR were all below the recommended critical thresholds. Crucially, the chi-square difference test for the strict invariant model yielded a non-significant result (*p* = 0.926), empirically confirming that imposing cross-cohort equality constraints on item residuals did not cause any statistical degradation in overall model fit. Therefore, the three-factor measurement model of PET, IM, and LE sequentially satisfied configural invariance, loading invariance, intercept invariance, and strict invariance across the two sample groups. These results indicate that the three core constructs possess good measurement comparability in the Shanghai and Kazakhstan samples, providing necessary measurement prerequisites for subsequent inter-group mean comparisons and multi-group structural path analyses.

### 4.2 Descriptive statistics, correlation analysis, and baseline comparison

After the measurement model achieved strict invariance, this study further compared the baseline levels of the two groups of core variables and investigated the bivariate correlation patterns within the full sample. In the full sample, the means for Perceived Educational Transformation (PET), Intrinsic Motivation (IM), and Learning Engagement (LE) were 3.451 (SD = 0.639), 3.543 (SD = 0.634), and 3.624 (SD = 0.608), respectively, all higher than the theoretical midpoint of 3.0 on the 5-point scale. From the descriptive results, the overall evaluations of university students from both cohorts regarding educational environment transformation, internal learning drive, and daily Learning Engagement were all at an upper-middle level.

The Pearson correlation analysis results are shown in Table 4. PET, IM, and LE all showed significant positive correlations with each other. Among them, PET was significantly positively correlated with IM (*r* = 0.371, *p* < 0.001), PET was significantly positively correlated with LE (*r* = 0.425, *p* < 0.001), and IM was also significantly positively correlated with LE (*r* = 0.398, *p* < 0.001). The three sets of correlation coefficients were all at a moderate level, and the maximum correlation coefficient was 0.425, showing no excessive overlap between the constructs, which provided a foundation for subsequent structural model analysis.

**Table 4:** Pearson correlation matrix (full sample, *N* = 1,309).

Variables	PET	IM	LE
PET	–	0.371^***^	0.425^***^
IM	0.371^***^	–	0.398^***^
LE	0.425^***^	0.398^***^	–

PET = Perceived Educational Transformation; IM = Intrinsic Motivation; LE = Learning Engagement. ^***^p < 0.001, two-tailed test.

After clarifying the overall correlation patterns, this study further used independent samples *t*-tests synthesizing composite scores to compare the baseline levels of PET, IM, and LE between university students from Shanghai and Kazakhstan, with detailed results in Table 5.

**Table 5:** Independent samples *t*-test results (*df* = 1,307).

Variable	Shanghai *M* (SD)	Kazakhstan *M* (SD)	*t*	*p*	Cohen’s *d*	Hypothesis and Conclusion
PET	3.514 (0.617)	3.378 (0.655)	3.855	<0.001^***^	0.214	H1a supported
IM	3.564 (0.660)	3.519 (0.602)	1.289	0.197	0.071	Generally consistent with H1b expectation
LE	3.632 (0.617)	3.614 (0.598)	0.539	0.590	0.030	Generally consistent with H1b expectation

PET = Perceived Educational Transformation; IM = Intrinsic Motivation; LE = Learning Engagement. ^***^p < 0.001; Cohen’s d interpretation reference: <0.20 is a trivial effect, 0.20–0.50 is a small effect.

The results indicate that the two sample groups present a specific pattern of baseline differences. In the dimension of Perceived Educational Transformation, the Shanghai sample was significantly higher than the Kazakhstan sample, and the inter-group difference reached a small effect level, thereby supporting H1a. However, similar inter-group differences did not appear in the dimensions of Intrinsic Motivation and Learning Engagement: neither test reached statistical significance. More importantly, the effect sizes for IM and LE were strictly within the trivial range (Cohen’s *d* < 0.10). While a non-significant *p*-value alone does not formally establish statistical equivalence, these negligible effect sizes practically indicate that the baseline motivational and engagement characteristics of the two groups are highly homologous, which is largely consistent with the expectation proposed by H1b.

Figure 3 illustrates the means and 95% confidence intervals of the Shanghai and Kazakhstan samples on PET, IM, and LE.

**Figure 3:**
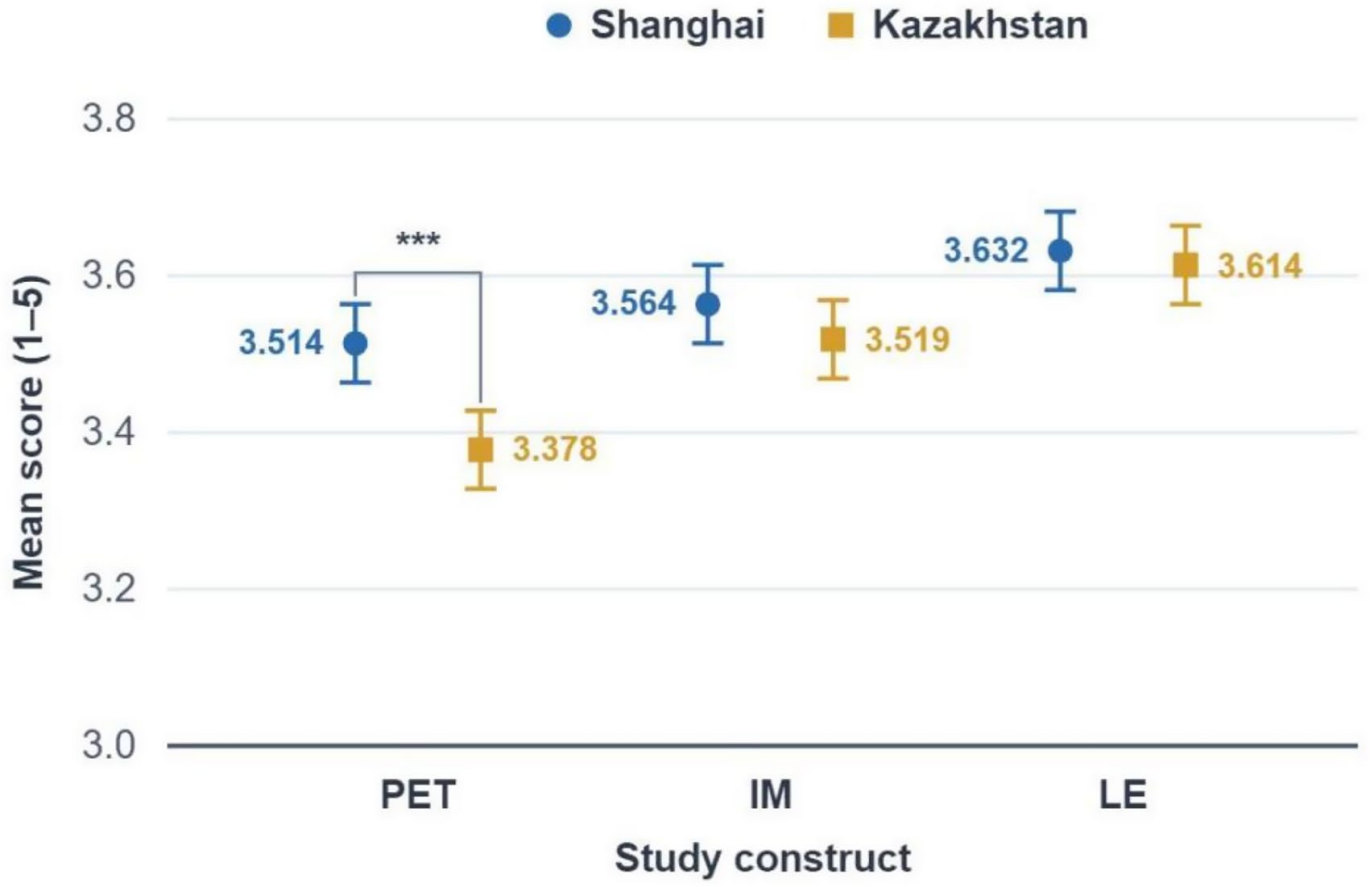
Mean comparison of PET, IM, and LE between Shanghai and Kazakhstan University Students. Error bars represent 95% confidence intervals. Connecting brackets indicate statistically significant mean differences between the two groups (^***^p < 0.001).

### 4.3 Indirect structural association testing

Building on the baseline findings, the analytical focus logically shifts to the internal structure of the co-variation associations among the variables. This study aims to examine whether the known overall positive association between PET and LE is partially realized through a systemic co-variation overlap with IM (Hypotheses H2 and H3). To this end, the study estimated the entire potential indirect pathway of PET → IM → LE within the Structural Equation Modeling (SEM) framework, using the Bootstrap resampling method (Hayes, 2017; 5,000 times, bias-corrected 95% confidence interval). The relevant path parameters are summarized in Table 6.

**Table 6:** Mediation Effect Testing Results (Full Sample, *N* = 1,309).

Path	Coefficient	*p*	Bootstrap 95% CI
a: PET → IM	0.369	<0.001^***^	[0.320, 0.419]
b: IM → LE (Controlling PET)	0.267	<0.001^***^	[0.215, 0.317]
c: PET → LE (Total effect)	0.405	<0.001^***^	[0.359, 0.450]
c’: PET → LE (Direct effect)	0.307	<0.001^***^	[0.257, 0.354]
ab: Indirect effect	0.098	–	[0.076, 0.122]
*R*^2^ (IM)	0.138		
*R*^2^ (LE)	0.248		

Bootstrap sampling 5,000 times; confidence intervals strictly excluding zero indicate a significant indirect effect; SEs for paths *a*, *b*, *c*, and *c*′ are based on OLS estimation; ^***^p < 0.001.

As a primary premise, at the full sample level, PET and LE showed a significant positive overall association (total effect *c* = 0.405, *p* < 0.001). This foundational result provided support for further conducting indirect effect analysis, thereby supporting Hypothesis H2.

After further introducing Intrinsic Motivation (IM) as an intermediate linking term, the path analysis showed: on the one hand, PET exhibited a significant positive co-variation association with IM (path *a* = 0.369, *p* < 0.001); on the other hand, holding PET constant, the active state of IM also maintained a significant synchronous association with LE (path *b* = 0.267, *p* < 0.001). Crucially, the indirect effect measurement results indicated that the indirect association of this structural framework was statistically significant (*ab* = 0.098, 95% CI [0.076, 0.122]). This clearly indicates that a considerable portion of perceived environmental transformation is statistically linked to final academic engagement behaviors through co-variation intertwining with students’ inner dynamics, explicitly supporting Hypothesis H3. Also, it is worth noting that since the direct association path between PET and LE (*c*′ = 0.307, *p* < 0.001) remained significant after adding IM, this internal model presents a structural form of partial associative covariation in a statistical sense.

Given the cross-sectional nature of the data, an alternative reverse sequence model (LE → IM → PET) was also exploratory tested. The alternative model yielded notably inferior conceptual coherence with fundamental SDT propositions. Therefore, while statistical bi-directionality cannot be definitively ruled out without longitudinal validation, the hypothesized PET → IM → LE sequence serves as the most theoretically sound associative framework for the current data. The currently equipped module contributed 13.8 and 24.8% of the explanatory power to the overall variance of IM and LE, respectively.

The indirect association model of Perceived Educational Transformation, Intrinsic Motivation, and Learning Engagement is shown in Figure 4.

**Figure 4:**
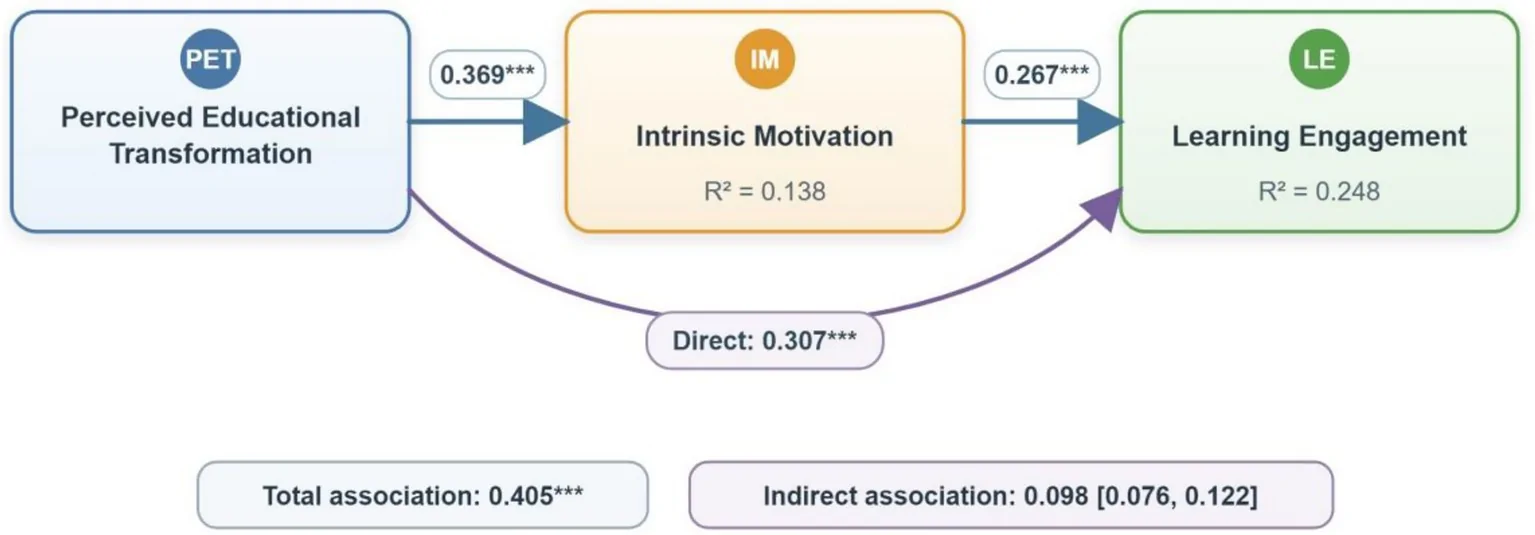
Mediation association model of Perceived Educational Transformation, Intrinsic Motivation, and Learning Engagement. Values in square brackets are bootstrap 95% confidence intervals; ^***^p < 0.001. Arrows indicate association directions based on theoretical settings, not representing that cross-sectional data has established causal relationships.

To rule out confounding variables stemming from potential biases in the resource baseline of the evaluated subjects, this analysis additionally incorporated the subjects’ family socioeconomic status (SES, based on the self-assessment indicator from Q10) as a covariate into the path framework to re-estimate the model. The comparison test showed excellent anti-interference characteristics; the variation range of all key path coefficients before and after adding the covariate was minute (range less than 0.001, for example, the indirect effect parameter remained steadfastly at 0.098). This further confirmed that the structural associations discovered in this paper exhibit an excellent level of robustness and were not systematically interfered with by socioeconomic difference groups.

### 4.4 Multi-group analysis: inter-group comparison of structural paths

Given the established strict measurement invariance, Multi-Group Structural Equation Modeling (MGSEM) was employed to separately estimate the structural paths in the Shanghai sample (*n* = 698) and the Kazakhstan sample (*n* = 611), in order to test whether the PET—IM—LE association structure varies by macro-national context. The results are shown in Table 7.

**Table 7:** Comparison of structural paths for Shanghai and Kazakhstan samples.

Path or indicator	Shanghai *B* (SE)	Shanghai *β*	Kazakhstan *B* (SE)	Kazakhstan *β*	Δ*B*	*z*	*p*
a: PET → IM	0.398(0.038)	0.372	0.340(0.035)	0.370	0.058	1.136	0.256
b: IM → LE (Controlling PET)	0.268(0.033)	0.287	0.263(0.038)	0.265	0.005	0.106	0.916
c′: PET→LE (Direct effect)	0.320(0.035)	0.321	0.299(0.035)	0.328	0.021	0.421	0.674
c: PET→LE (Total effect)	0.427(0.034)	0.427	0.389(0.033)	0.426	0.038	0.799	0.425
ab: Indirect effect	0.107 [0.075, 0.142]	0.107	0.089 [0.059, 0.122]	0.098	0.017	–	95%CIΔ = [−0.028, 0.063]
*R*^2^ (IM)	0.138	–	0.137	–	–	–	–
*R*^2^ (LE)	0.254	–	0.242	–	–	–	–

All paths within both groups reached statistical significance (*p* < 0.001). B is the unstandardized coefficient; *β* is the standardized coefficient; values in parentheses are standard errors. The confidence interval for indirect effect *ab* is calculated based on 5,000 Bootstrap samplings; Δ*B* is calculated as “Shanghai coefficient minus Kazakhstan coefficient”; 95%CIΔ is the Bootstrap confidence interval of the difference in indirect effects, containing 0 means the inter-group difference is not significant.

The grouped estimation results showed that the PET—IM—LE association was supported in both samples: the main structural paths of both groups showed significant positive associations, the Bootstrap 95% confidence intervals of IM’s indirect association with PET and LE did not include 0, and the direct association between PET and LE remained significant after controlling for IM, indicating that both samples exhibited the structural characteristics of partial statistical indirect association.

However, the inter-group differences for each path and the indirect effect did not reach statistical significance. Therefore, this study found no evidence that the macro-national context exerted a significant moderating effect on the PET—IM—LE association structure; H4 was not supported. To ensure that failing to reject the null hypothesis was not an artifact of an underpowered design, a post-hoc statistical power analysis was evaluated. The results confirmed that with the current substantial sub-sample sizes (*n* > 600 per group), the test possessed robust power (1 − *β* > 0.80) to detect even moderate structural path differences between cohorts. Consequently, the convergence of these paths strongly reflects genuine structural consistency across both educational contexts rather than a Type II error.

The comparison of structural path coefficients between the Shanghai and Kazakhstan samples is shown in Figure 5.

**Figure 5:**
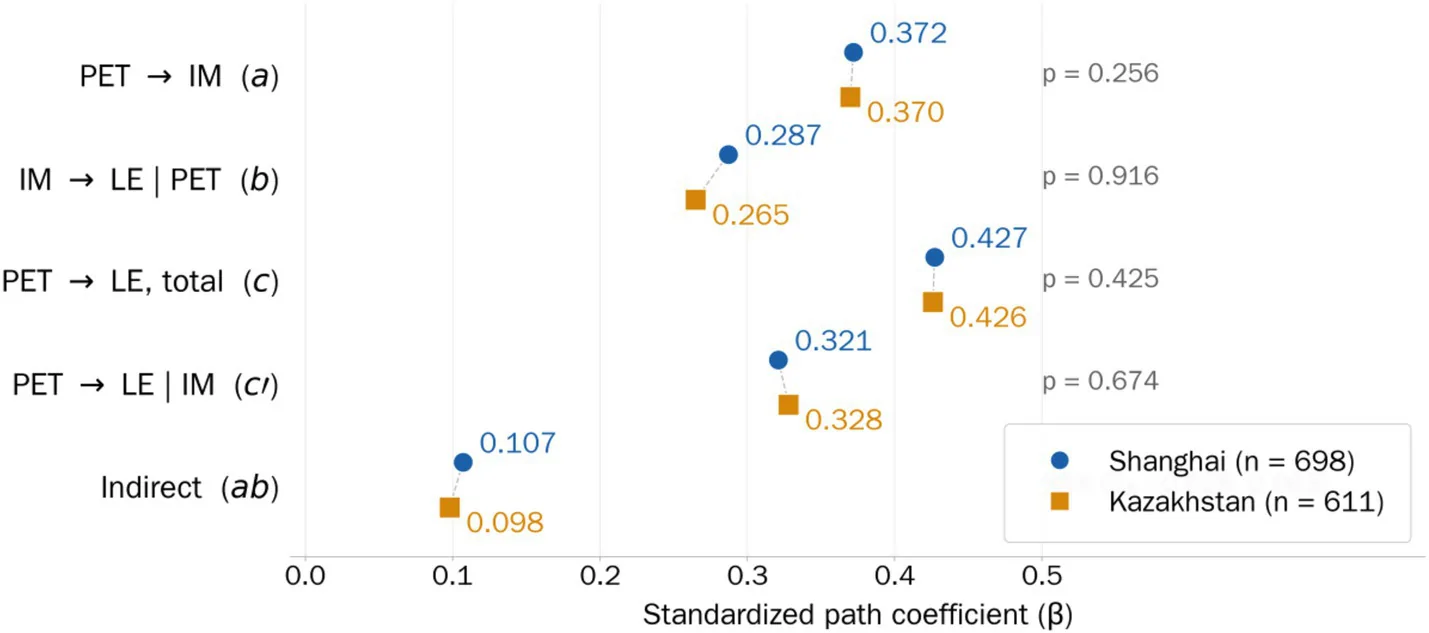
Comparison of structural path coefficients (*β*) in Shanghai and Kazakhstan samples. Dots denote the Shanghai sample, squares denote the Kazakhstan sample, and dashed lines connect the estimates of the two groups for the same path. Values in the figure are standardized path coefficients; the inter-group differences of all paths and indirect effects did not reach statistical significance (*p* > 0.05). The bootstrap 95% confidence interval for the difference in indirect effects is [−0.028, 0.063], containing 0. Exact inter-group test results are shown in Table 7.

### 4.5 Demographic heterogeneity testing

Supplementary independent samples *t*-tests were conducted to examine demographic heterogeneity (gender and educational level) across the core variables. The test results are summarized in Tables 8, 9.

**Table 8:** Supplementary inter-group comparisons by gender (male vs. female).

Variable	Sample	Male *M* (SD)	Female *M* (SD)	*t*	*p*	Cohen’s *d*
PET	Full Sample	3.417 (0.653)	3.470 (0.618)	−1.479	0.139	−0.083
PET	Shanghai	3.482 (0.638)	3.523 (0.590)	−0.872	0.383	−0.067
PET	Kazakhstan	3.348 (0.662)	3.405 (0.645)	−1.064	0.288	−0.087
IM	Full Sample	3.556 (0.613)	3.529 (0.646)	0.786	0.432	0.044
IM	Shanghai	3.569 (0.642)	3.561 (0.677)	0.160	0.873	0.012
IM	Kazakhstan	3.543 (0.582)	3.490 (0.605)	1.096	0.274	0.089
LE	Full Sample	3.636 (0.609)	3.607 (0.610)	0.857	0.392	0.048
LE	Shanghai	3.642 (0.613)	3.615 (0.624)	0.570	0.569	0.044
LE	Kazakhstan	3.629 (0.606)	3.597 (0.594)	0.668	0.505	0.055

Gender only included valid responses of male (Q6 = 1) and female (Q6 = 2). All comparisons were not significant (*p* > 0.05), and effect sizes were within the trivial range.

**Table 9:** Supplementary inter-group comparisons by educational level (undergraduates vs. graduate students).

Variable	Sample	Undergraduate n	Undergraduate M (SD)	Graduate n	Graduate M (SD)	t	*p*	Cohen’s *d*
PET	Full Sample	978	3.440 (0.632)	331	3.483 (0.659)	−1.073	0.283	−0.068
PET	Shanghai	524	3.490 (0.622)	174	3.586 (0.601)	−1.780	0.076	−0.156
PET	Kazakhstan	454	3.382 (0.639)	157	3.369 (0.702)	0.201	0.841	0.019
IM	Full Sample	978	3.547 (0.627)	331	3.533 (0.654)	0.330	0.742	0.021
IM	Shanghai	524	3.542 (0.649)	174	3.632 (0.691)	−1.567	0.118	−0.137
IM	Kazakhstan	454	3.552 (0.602)	157	3.424 (0.592)	2.314	0.021	0.214
LE	Full Sample	978	3.615 (0.604)	331	3.652 (0.618)	−0.967	0.334	−0.062
LE	Shanghai	524	3.609 (0.613)	174	3.704 (0.625)	−1.762	0.079	−0.154
LE	Kazakhstan	454	3.621 (0.594)	157	3.594 (0.608)	0.482	0.630	0.045

Educational level grouping: Undergraduates (Q8 = 1–4) vs. combined Master’s/Doctoral graduate students (Q8 = 5–7). ^*^p < 0.05. This analysis is an exploratory supplementary test without multiple comparison correction, and results interpretation should be correspondingly conservative.

In the gender dimension, whether at the full sample level or down to the specific subgroupings of Shanghai and Kazakhstan, the means on PET, IM, and LE for male and female groups showed no statistically significant differences, and all effect sizes were within a trivial range (∣*d*∣ < 0.10). This indicates that in the dual-national context of this study, the gender variable did not constitute a systemic dividing line for the distribution of the three core variables.

In the comparison of educational levels (undergraduates vs. combined graduate students), neither PET nor LE showed statistically significant differences in the full sample or the two group samples. It is noteworthy that in the Kazakhstan sample, the mean of Intrinsic Motivation for undergraduates (*M* = 3.552, SD = 0.602) was slightly higher than that for graduate students (*M* = 3.424, SD = 0.592), and the difference reached statistical significance, *t*(609) = 2.314, *p* = 0.021, *d* = 0.214, which is a small effect. However, considering that this was an isolated exploratory finding emerging without family-wise error correction (such as Bonferroni adjustments) and was not replicated in the full or Shanghai samples, it should be interpreted with high caution. The direction also contradicted the conventional expectation that “graduate students have deeper engagement.” Therefore, this isolated significant result may be an incidental finding under conditions of multiple comparisons, and caution should be maintained when interpreting it.

Taken together, neither gender nor educational level presented systematic inter-group differences on the three core variables, suggesting that the main structural associations established in this paper were not subject to prominent demographic confounding.

## 5 Discussion

Against the backdrop of the macro-educational system’s evolution, this study isolated a micro-adaptive associating external environmental perception through psychological arousal to specific learning behaviors. Through a comparative analysis of 1,309 university students from the Shanghai sample and Kazakhstan, the data revealed a psychological co-variation pattern transcending specific institutional differences while simultaneously delineating the boundaries of contextual impacts.

### 5.1 The nested co-variation framework of environmental perception and Intrinsic Motivation

Exploring how macro-policies are reflected in individual proximal experiences is an area rarely touched upon by traditional top-down educational assessments. This study verified a robust positive association between Perceived Educational Transformation (PET)—operationalized strictly as micro-classroom perceptions rather than macro-policy proxies—and Learning Engagement (LE), further revealing the bridging associative role of Intrinsic Motivation (IM) in between. The establishment of this indirect structural association (*ab* = 0.098) provided empirical evidence for the applicability of Self-Determination Theory (SDT) in the context of higher education transformation (Bureau et al., 2022). When students capture localized environmental signals that encourage independent thinking, tolerate trial-and-error, and diversify evaluations, this atmosphere, which aligns with autonomy-supportive needs, can accompany an active state of students’ exploratory desires; and the enrichment of these internal psychological resources further accompanies higher levels of concentration and behavioral resilience exhibited by individuals in their daily academic routines (Standage et al., 2025; Wang et al., 2024). Furthermore, this structural linkage aligns seamlessly with recent empirical advancements suggesting that inclusive, autonomy-conducive micro-environments are pivotal in translating affective and motivational resources into sustained, high-quality engagement (Tian et al., 2026; Yang, J. et al., 2025).

### 5.2 Cross-cultural mapping: structural stability across distinct cohorts

Placing the cross-sectional data within the vastly different educational evolutionary trajectories of the Shanghai and Kazakhstani cohorts presented deeper theoretical findings. In the baseline test, Shanghai students reported a significantly higher Perceived Educational Transformation (supporting hypothesis H1a). Considering that Shanghai is at the forefront of China’s educational reforms, its classrooms have arguably released denser transformation signals in terms of discourse systems and practice iterations, and this result meets expectations (Luo, 2023).

However, an empirically fascinating phenomenon worthy of further discussion is that: although the Shanghai sample reported a higher localized perception of educational transformation, the negligible effect sizes (*d* < 0.10) for Intrinsic Motivation (IM) and Learning Engagement (LE) indicated highly homologous baseline motivational states across the two cohorts, which is largely consistent with the expectation proposed by H1b. Rather than speculating on unmeasured post-hoc sociological mechanisms—such as overarching competitive strain or graduate screening anxiety—which cannot be empirically verified herein, this asynchrony highlights a rigorous theoretical boundary: heightened micro-perceptions of pedagogical reform do not automatically confer proportional, absolute increases in Intrinsic Motivation. It suggests that while supportive micro-environments are crucial structurally, terminal motivational baselines remain complex products heavily anchored by broader, yet-to-be-measured systemic realities (Deng et al., 2024; Jiang and Wang, 2026).

In the equivalence testing of the association structure, the data also exhibited highly resilient cross-contextual consistency. The PET → IM → LE mechanism was not only established independently in both cohorts, but the inter-group differences of core path parameters were not statistically significant (with robust post-hoc power confirming no Type II error; H4 was not supported). Crucially, while a lack of significant group differences does not statistically equate to absolute cross-cultural “universality,” it strongly denotes that whether in an East Asian system that has undergone high-performance competition or in a Central Asian country advancing international integration, the psychological generation mechanism based on autonomy support associating with internal dynamics shares the same underlying logic and possesses structural stability (King and McInerney, 2014).

Nevertheless, to elevate the theoretical depth of this cross-cultural comparison, a distinction must be explicitly drawn between statistical measurement invariance and the socio-cultural projections of “transformation.” While the PET scale operates with semantic equivalence across both samples in assessing autonomy-supportive features, the substantive macro-historical referents behind these perceptions likely differ qualitatively. For the Chinese cohort, transformation is primarily experienced as an endogenous pedagogical shift—moving from a rigid test-oriented paradigm toward a competency-based model. In contrast, for the Kazakhstani cohort, these perceived changes are deeply entangled with the exogenous transition toward global integration and Western higher education standardization (such as the Bologna Process). Recognizing this qualitative divergence adds crucial nuance to our findings: it reveals a fascinating academic reality where heterogeneous macro-sociological triggers (endogenous de-emphasizing of exams vs. exogenous international integration) ultimately converge to facilitate a homogeneous micro-psychological mechanism—activating Intrinsic Motivation via autonomy support.

### 5.3 Heterogeneity of micro-ecology: localized demographic variations

After accounting for cohort-level differences, the demographic variations within the micro-mechanisms in the micro-mechanisms exhibited steady homogeneit. The gender dimension showed no statistical differentiation between male and female groups in terms of perceptual acuity and motivational activation (all ∣*d*∣ < 0.10).

In the comparison of educational levels, neither PET nor LE showed systematic effects. The only isolated result reaching statistical significance appeared in the IM dimension for the Kazakhstan sample, where undergraduates’ Intrinsic Motivation was slightly higher than that of graduate students (*d* = 0.214). However, because this isolated variance emerged without family-wise error correction and contradicted both conventional expectations and the broader full-sample patterns, it is most conservatively interpreted as an incidental statistical artifact rather than a robust theoretical divergence. Overall, demographic variables did not constitute systematic interference with the main structural associations of this study.

### 5.4 Limitations and future prospects

Constrained by the research design, this study has several limitations at the evidence deduction and extrapolation level that must be squarely faced.

First, the temporal constraints of the cross-sectional design. This study relied on cross-sectional observations to establish SEM models, which in essence still belonged to the demarcation of co-variation relationships among statistical variables. Although the “perception → motivation → engagement” path mathematically fit the data and possessed the *a priori* support of SDT theory, temporal causality or the definitive exclusion of recursive relationships cannot be inferred in the absence of time variables. Future research urgently needs to introduce a cross-lagged longitudinal tracking design to capture the dynamic sequence of motivation evolution and engagement generation.

Second, the variance interference risk of single-source measurement. Although this study eliminated severe Common Method Bias (CMB) threats by pre-filling informed consents, embedding attention tests, evaluating via Harman’s single factor, and Common Latent Factor (CLF) validation, relying on individual subjective assessments may still underestimate the confounding interference of external systemic factors. If future research can combine multi-source data such as teacher evaluations and classroom observation evaluations to implement data triangulation, it will be able to further break through measurement bottlenecks.

Third, the localized nature of the motivation dimension. This study focused on the pure state of internal drive (IM), but the motivational structure in the actual academic ecology is far more complex. Constructing a pluralistic competitive model that simultaneously accommodates internal momentum and external regulation (e.g., integrated regulation, introjected regulation) may be closer to the real profile of students’ academic adaptation.

Fourth, limitations of sample representativeness. This study utilized purposive samples from cooperative universities in Shanghai and Kazakhstan, rather than national probability samples of the two countries. Shanghai possesses a higher density of educational resources and a stronger intensity of reform promotion; extreme caution should be maintained when extending the relevant findings to other regions in China or broader Central Asian populations.

## 6 Conclusion and implications

### 6.1 Research conclusion

The paradigm shift of the macro-educational system ultimately depends on the psychological acceptance and adaptive state of micro-learners to manifest as measurable pedagogical realities. This study confirmed that there is a significant positive association between Perceived Educational Transformation—anchored strictly in proximal classroom experiences—and Learning Engagement, Crucially, this association can be explained at the statistical level by the partial indirect associative mechanism of Intrinsic Motivation; the co-variation relationship between Perceived Educational Transformation and deep-level Learning Engagement is structurally closely linked to the effective activation of the individual’s internal motivation resources. This initially verified statistical associative framework of “environmental transformation perception—motivation activation—Learning Engagement” exhibited excellent structural stability in the vastly different academic ecological contexts of the Shanghai cohort and Kazakhstan, suggesting a shared underlying psychological logic across these distinct groups rather than absolute macro-level universality.

### 6.2 Theoretical and practical implications

At the theoretical level, this study built an empirical bridge between macro-educational evolution discourses and micro-educational psychology frameworks, clarifying the psychological fact that macro-reforms do not mechanically dictate student outcomes, but rather must pass through the media of proximal individual perception and motivation to present associations.

In the practical dimension, the research results conveyed specific signals to educational administrators: intending to improve the learning state by imposing performance assessments external to tasks may have extremely limited marginal utility. High-level ecological reshaping at the systemic level must avoid hovering merely at the level of abstract advocacy and instead sink solidly into the daily operations of micro-contexts—exploring how to change questioning methods in classrooms, expand substantial trial-and-error spaces, and grant students psychological safety on non-standardized evaluation indicators. The ultimate leap in the quality of educational evaluation will inevitably return to how to more meticulously care for and support the internal self-driven thirst for knowledge of every independent learner.

## Data Availability

The original contributions presented in the study are included in the article/Supplementary material, further inquiries can be directed to the corresponding author.

## References

[ref1] Assefa Y. Tilwani S. A. Moges B. T. Majeed H. (2025). Unpacking student engagement and its mediating role in predicting the relationship between self-determination and academic satisfaction among undergraduate students in higher education. Soc. Sci. Humanit. Open 12:101797. doi: 10.1016/j.ssaho.2025.101797

[ref2] Bing D. (2026). Teachers’ emotional support and college students’ adaptation: the mediating roles of intrinsic goals and psychological need satisfaction. Front. Psychol. 17:1792547. doi: 10.3389/fpsyg.2026.1792547, 42039005 PMC13106458

[ref3] Bridges D. (eds.) (2014). Education Reform and Internationalisation: The Case of School Reform in Kazakhstan. Cambridge: Cambridge University Press.

[ref4] Bureau J. S. Howard J. L. Chong J. X. Guay F. (2022). Pathways to student motivation: a meta-analysis of antecedents of autonomous and controlled motivations. Rev. Educ. Res. 92, 46–72. doi: 10.3102/00346543211042426, 35330866 PMC8935530

[ref5] Chen F. F. (2007). Sensitivity of goodness of fit indexes to lack of measurement invariance. Struct. Equ. Model. A Multidiscip. J. 14, 464–504. doi: 10.1080/10705510701301834

[ref6] Cheung G. W. Rensvold R. B. (2002). Evaluating goodness-of-fit indexes for testing measurement invariance. Struct. Equ. Model. 9, 233–255. doi: 10.1207/S15328007SEM0902_5

[ref7] Deci E. L. Ryan R. M. (2013). Intrinsic Motivation and Self-Determination in Human Behavior. New York: Springer Science & Business Media.

[ref8] Deng L. Wu Y. Chen L. Peng Z. (2024). Pursuing competencies’ or ‘pursuing scores’? High school teachers’ perceptions and practices of competency-based education reform in China. Teach. Teach. Educ. 141:104510. doi: 10.1016/j.tate.2024.104510

[ref9] Duman S. (2024). “Education reforms in Kazakhstan: international integration and nationalization efforts,” in Global Agendas and Education Reforms: A Comparative Study, (Singapore: Springer Nature Singapore), 41–67.

[ref10] Fornell C. Larcker D. F. (1981). Evaluating structural equation models with unobservable variables and measurement error. J. Mark. Res. 18, 39–50. doi: 10.1177/002224378101800104

[ref11] Hair J. F. Jr. Black W. C. Babin B. J. Anderson R. E. (2010). Multivariate Data Analysis. 7th Edn. London: Pearson.

[ref12] Hayes A. F. (2017). Introduction to Mediation, Moderation, and Conditional Process Analysis: A Regression-Based Approach. New York: Guilford Publications.

[ref13] Henseler J. Ringle C. M. Sarstedt M. (2015). A new criterion for assessing discriminant validity in variance-based structural equation modeling. J. Acad. Mark. Sci. 43, 115–135. doi: 10.1007/s11747-014-0403-8

[ref14] Jiang H. Wang Y. (2026). Academic self-efficacy and learning engagement among law-English dual degree students: the mediating role of professional identity under the foreign-related rule of law orientation. Eur. J. Educ. 61:e70860. doi: 10.1111/ejed.70860

[ref15] King R. B. McInerney D. M. (2014). Culture’s consequences on student motivation: capturing cross-cultural universality and variability through personal investment theory. Educ. Psychol. 49, 175–198. doi: 10.1080/00461520.2014.926813

[ref16] Kuzhabekova A. (2025). Uncovering the potential of equity as a transformative force in higher education reform in post-Nazarbayev Kazakhstan. Central Asian Survey, 1–21. doi: 10.1080/02634937.2025.2566291, 37339054

[ref17] Li J. (2012). Cultural Foundations of Learning: East and West. Cambridge: Cambridge University Press.

[ref18] Lo N. P. K. (2024). Cross-cultural comparative analysis of student motivation and autonomy in learning: perspectives from Hong Kong and the United Kingdom. Front. Educ. 9:1393968. doi: 10.3389/feduc.2024.1393968

[ref19] Luo S. (2023). The current landscape and future direction of curriculum reform in China. Future Educ. Res. 1, 5–16. doi: 10.1002/fer3.8

[ref20] Narbaev T. Amirbekova D. Bakdaulet A. (2025). A decade of transformation in higher education and science in Kazakhstan: a literature and scientometric review of national projects and research trends. Publica 13:35. doi: 10.3390/publications13030035

[ref21] OECD (2013). PISA 2012 Results: Ready to Learn (Volume III): Students’ Engagement, Drive and Self-Beliefs. Paris: OECD Publishing.

[ref22] OECD (2017). PISA 2015 Results (Volume III): Students’ Well-Being. Paris: OECD Publishing.

[ref23] OECD (2019). PISA 2018 Results (Volume III): What School Life Means for Students’ Lives. Paris: OECD Publishing.

[ref24] OECD (2023). PISA 2022 Results (Volume I): The State of Learning and Equity in Education. Paris: OECD Publishing.

[ref25] Podsakoff P. M. MacKenzie S. B. Lee J. Y. Podsakoff N. P. (2003). Common method biases in behavioral research: a critical review of the literature and recommended remedies. J. Appl. Psychol. 88, 879–903. doi: 10.1037/0021-9010.88.5.879, 14516251

[ref26] Ryan R. M. Deci E. L. (2000). Intrinsic and extrinsic motivations: classic definitions and new directions. Contemp. Educ. Psychol. 25, 54–67. doi: 10.1006/ceps.1999.1020, 10620381

[ref27] Ryan R. M. Deci E. L. (2020). Intrinsic and extrinsic motivation from a self-determination theory perspective: definitions, theory, practices, and future directions. Contemp. Educ. Psychol. 61:101860. doi: 10.1016/j.cedpsych.2020.101860

[ref28] Schaufeli W. B. Martínez I. M. Marques Pinto A. Salanova M. Bakker A. B. (2002). Burnout and engagement in university students: a cross-national study. J. Cross-Cult. Psychol. 33, 464–481. doi: 10.1177/0022022102033005003

[ref29] Seitzer H. Niemann D. Martens K. (2021). Placing PISA in perspective: the OECD’S multi-centric view on education. Glob. Soc. Educ. 19, 198–212. doi: 10.1080/14767724.2021.1878017

[ref30] Silova I. Steiner-Khamsi G. (eds.) (2008). How NGOs React: Globalization and Education Reform in the Caucasus, Central Asia and Mongolia. Bloomfield, CT: Kumarian Press.

[ref31] Standage M. Ryan R. M. Curran T. (2025). “Self-determination theory applied to physical education: the role of self-regulatory processes in facilitating high-quality student motivation, engagement, and well-being,” in Motivation in Physical Education, (Cham: Springer Nature Switzerland), 29–51.

[ref32] Taylor P. C. Fraser B. J. Fisher D. L. (1997). Monitoring constructivist classroom learning environments. Int. J. Educ. Res. 27, 293–302. doi: 10.1016/S0883-0355(97)90011-2

[ref33] Tian W. Wang Y. Wang Y. (2026). Affective processes in perceived inclusive EFL classrooms: linking self-efficacy, engagement, resilience, and attitudes towards generative AI. Acta Psychol. 267:107103. doi: 10.1016/j.actpsy.2026.107103 42176447

[ref34] Vallerand R. J. Pelletier L. G. Blais M. R. Brière N. M. Senécal C. Vallières E. F. (1992). The academic motivation scale: a measure of intrinsic, extrinsic, and amotivation in education. Educ. Psychol. Meas. 52, 1003–1017. doi: 10.1177/0013164492052004025

[ref35] Wang Y. S. Xin Y. Chen L. (2024). Navigating the emotional landscape: insights into resilience, engagement, and burnout among Chinese high school English as a foreign language learners. Learn. Motiv. 86:101978. doi: 10.1016/j.lmot.2024.101978

[ref36] Wei X. Ming Y. Jing J. Hao J. (2025). The transformation of China’s education system: between Confucian traditions and global modernization. Int. J. Educ. Soc. Sci. Stud. 1, 42–60. doi: 10.60153/ijesss.v1i2.192, 41337989

[ref37] Williams G. C. Deci E. L. (1996). Internalization of biopsychosocial values by medical students: a test of self-determination theory. J. Pers. Soc. Psychol. 70, 767–779. doi: 10.1037/0022-3514.70.4.767, 8636897

[ref38] Yakavets N. Winter L. Malone K. Zhontayeva Z. Khamidulina Z. (2023). Educational reform and teachers’ agency in reconstructing pedagogical practices in Kazakhstan. J. Educ. Change 24, 727–757. doi: 10.1007/s10833-022-09463-5

[ref39] Yang J. Chen Y. Wang Y. (2025). Exploring the interplay of motivation, engagement, and critical thinking among EFL learners: evidence from structural equation modelling. Eur. J. Educ. 60:e70187. doi: 10.1111/ejed.70187

[ref40] Yang Y. Chen J. Zhuang X. (2025). Self-determination theory and the influence of social support, self-regulated learning, and flow experience on student learning engagement in self-directed e-learning. Front. Psychol. 16:1545980. doi: 10.3389/fpsyg.2025.1545980, 40207130 PMC11979257

[ref41] Zhang Y. Yang X. Sun X. Kaiser G. (2023). The reciprocal relationship among Chinese senior secondary students’ intrinsic and extrinsic motivation and cognitive engagement in learning mathematics: a three-wave longitudinal study. ZDM—Math. Educ. 55, 399–412. doi: 10.1007/s11858-022-01465-0

