# Supplementary material for: Evolution of the educational system and micro-psychological mechanisms: a comparative study of academic motivation and engagement in Shanghai, China and Kazakhstan
Source: Front Psychol. 2026 Oct 28;17:2005404. doi: 10.3389/fpsyg.2026.2005404 (PMC13751599; doi:10.3389/fpsyg.2026.2005404)
Supplement: Supplementary file 1 [file Data_Sheet_1.zip › Supplementary_Material.docx]

## **Appendix A: Multilingual Questionnaire**

## English Reference Version of the Questionnaire

### A. Participant Information and Consent

Dear Student,

We are conducting an academic survey on university students’ learning experiences. The purpose of this study is to understand students’ intrinsic motivation, learning engagement, and perceptions of current university education under different educational contexts. This survey is intended only for currently enrolled university students aged 18 or above, and it will take approximately 6-8 minutes to complete.

This survey is anonymous. All data will be used for academic research only, and no personally identifiable information such as your name or student ID will be collected. Your participation is entirely voluntary, and you may stop answering at any time without any negative consequences.

**Ethics Information**

Participation in this study is entirely voluntary.

You may refuse to participate or withdraw at any time.

The survey is anonymous.

All data will be used only for academic purposes and analyzed in aggregate form.

This study involves minimal risk.

If any question causes discomfort, you may skip it or stop participating.

### Consent Item

**1. Have you read the information above and voluntarily agreed to participate in this study?**
1 = I agree to participate
2 = I do not agree to participate

### B. Screening Items

**2. Are you currently an enrolled university student?**
(undergraduate, master’s, or doctoral student)
1 = Yes
2 = No

**3. Are you 18 years old or above?**
1 = Yes
2 = No

**4. Where is your current university located?**
1 = Shanghai, China
2 = Kazakhstan
3 = Other country or region

### C. Demographic Information

**5. What is your current student status?**
1 = Undergraduate student
2 = Master’s student
3 = Doctoral student

**6. What is your gender?**
1 = Male
2 = Female
3 = Other
4 = Prefer not to say

**7. What is your age?**

**8. What is your current year of study?**
1 = First year undergraduate
2 = Second year undergraduate
3 = Third year undergraduate
4 = Fourth year undergraduate
5 = First year master’s
6 = Second year master’s or above
7 = Doctoral level

**9. Which category best describes your major?**
1 = Humanities and social sciences
2 = Science and engineering
3 = Medicine and health sciences
4 = Education
5 = Arts and sports
6 = Other

**10. How would you rate your family’s current economic status compared with others in your local area?**
1 = Far below average
2 = Slightly below average
3 = Average
4 = Slightly above average
5 = Far above average

**11. What is your father’s highest level of education?**
1 = Junior secondary school or below
2 = Senior high school or vocational school
3 = Junior college
4 = Bachelor’s degree
5 = Master’s degree or above
6 = Not sure / prefer not to say

**12. What is your mother’s highest level of education?**
1 = Junior secondary school or below
2 = Senior high school or vocational school
3 = Junior college
4 = Bachelor’s degree
5 = Master’s degree or above
6 = Not sure / prefer not to say

**13. How would you evaluate your current academic performance overall?**
1 = Very poor
2 = Poor
3 = Average
4 = Good
5 = Very good

### D. Perceived Educational Transition Scale

**Instruction.**
The following items are about your perceptions of current university education. Please choose the option that best matches your actual experience.

**Response options for Items 14.1-14.8**
1 = Strongly disagree
2 = Disagree
3 = Neutral
4 = Agree
5 = Strongly agree

**14. In my university learning experience…**

**14.1** Instructors often encourage us to express views that differ from standard answers.
**14.2** Classroom learning emphasizes applying knowledge to solve real-world problems.
**14.3** Courses place importance on collaboration, communication, and expression skills.
**14.4** Instructors encourage us to think independently rather than merely memorize knowledge points.
**14.5** Course assessment mainly depends on whether students remember a large number of facts and standard answers.
**14.6** Learning in my major emphasizes exam results more than understanding and application.
**14.7** In class, trying different approaches is usually encouraged.
**14.8** Making mistakes is usually regarded as part of learning and improvement.

### E. Intrinsic Motivation Scale

**Instruction.**
The following items are intended to understand why you are willing to keep engaging in university learning. Please respond according to your actual situation.

**Response options for Items 15.1-15.8**
1 = Strongly untrue of me
2 = Mostly untrue of me
3 = Neither true nor untrue
4 = Mostly true of me
5 = Strongly true of me

**15. I am willing to invest in university learning because…**

**15.1** I enjoy the pleasure of discovering new knowledge and new ideas.
**15.2** Understanding complex problems gives me a sense of satisfaction.
**15.3** Solving difficult learning problems gives me a sense of achievement.
**15.4** Learning itself is interesting to me.
**15.5** I enjoy exploring the principles and logic behind course content.
**15.6** I feel happy when I understand a concept that I did not understand before.
**15.7** Even without external rewards, I am willing to study content that interests me.
**15.8** Continuous learning allows me to feel personal growth.

### F. Learning Engagement Scale

**Instruction.**
The following items are about your engagement in daily learning. Please choose the option that best reflects your actual experience.

**Response options for Items 16.1-16.9**
1 = Never
2 = Rarely
3 = Sometimes
4 = Often
5 = Always

**16. During the learning process…**

**16.1** I feel full of energy.
**16.2** When I think about starting a day of study, I usually feel motivated to get going.
**16.3** I feel that what I study is meaningful.
**16.4** I am enthusiastic about what I am studying.
**16.5** I am often able to stay focused for a long time while studying.
**16.6** When I study seriously, time often passes quickly.
**16.7** Even when I have many learning tasks, I am willing to persist and complete them.
**16.8** I am able to immerse myself in my current learning activities.
**16.9** Learning stimulates my interest and inspiration.

### G. Attention Check Item

**17. To ensure data quality, please select “Mostly true of me” for this item.**
1 = Strongly untrue of me
2 = Mostly untrue of me
3 = Neither true nor untrue
4 = Mostly true of me
5 = Strongly true of me

### H. Supplementary Questions

**18. Overall, which orientation do you think current university education is closer to?**
1 = More focused on knowledge memorization and exam performance
2 = Roughly balanced between the two
3 = More focused on competence development and comprehensive qualities

**19. In your opinion, which factors most enhance your learning motivation in university?**
(Multiple choices allowed)
1 = Course content has real-life relevance
2 = Instructors encourage independent thinking
3 = Assessment methods are more diverse
4 = Learning content is challenging
5 = Peer collaboration and discussion
6 = Future employment or further study needs
7 = Personal interest
8 = Other: ____

# **Supplementary Material 2**

## Chinese-Language Questionnaire Used for the Shanghai Sample

**Note.**
The following instrument presents the Chinese-language version of the questionnaire administered to the Shanghai sample. All responses were collected anonymously and used only for academic research purposes.

### A. 参与者说明与知情同意

尊敬的同学，您好！

我们正在开展一项关于大学生学习体验的学术研究，旨在了解不同教育背景下大学生的学习动机、学习投入以及对当前大学教育方式的感受。本研究仅面向18周岁及以上的在校大学生，预计填写时间约6-8分钟。

本问卷采用匿名方式，所有数据仅用于学术研究，不收集您的姓名、学号等可识别个人身份的信息。您的参与完全出于自愿，您可以在任何时候停止作答，而不会受到任何不利影响。

**伦理说明**

参与本研究完全自愿

您可以在任何时候拒绝参与或退出作答

本问卷匿名填写

所有数据仅用于学术研究，并以汇总形式进行分析

本研究属于低风险研究

如个别题目让您感到不适，您可以跳过或退出问卷

### Consent Item

**1. 您是否已阅读上述说明，并自愿参加本研究？**
1 = 我同意参加
2 = 我不同意参加

### B. 筛选题

**2. 您目前是否为在校大学生（含本科生、硕士研究生、博士研究生）？**
1 = 是
2 = 否

**3. 您是否已满18周岁？**
1 = 是
2 = 否

**4. 您当前就读高校所在地区为：**
1 = 中国上海
2 = 哈萨克斯坦
3 = 其他国家/地区

### C. 基本信息

**5. 您目前的学习身份是：**
1 = 本科生
2 = 硕士研究生
3 = 博士研究生

**6. 您的性别是：**
1 = 男
2 = 女
3 = 其他
4 = 不愿透露

**7. 您的年龄是：**

**8. 您目前所在年级是：**
1 = 大一
2 = 大二
3 = 大三
4 = 大四
5 = 硕士一年级
6 = 硕士二年级及以上
7 = 博士阶段

**9. 您的专业类别更接近：**
1 = 人文社科类
2 = 理工类
3 = 医学健康类
4 = 教育学类
5 = 艺术体育类
6 = 其他

**10. 您认为自己家庭目前的经济状况在当地属于：**
1 = 明显低于平均水平
2 = 略低于平均水平
3 = 平均水平
4 = 略高于平均水平
5 = 明显高于平均水平

**11. 您父亲的最高受教育程度是：**
1 = 初中及以下
2 = 高中/中专
3 = 大专
4 = 本科
5 = 硕士及以上
6 = 不清楚/不便回答

**12. 您母亲的最高受教育程度是：**
1 = 初中及以下
2 = 高中/中专
3 = 大专
4 = 本科
5 = 硕士及以上
6 = 不清楚/不便回答

**13. 您对自己目前学业表现的总体评价是：**
1 = 很不理想
2 = 不太理想
3 = 一般
4 = 较好
5 = 很好

### D. 教育转型感知量表

**说明：**
以下题目旨在了解您对当前大学教育方式的感受。请根据您的真实体验选择最符合的一项。

**Response options for Items 14.1-14.8**
1 = 非常不同意
2 = 不同意
3 = 一般
4 = 同意
5 = 非常同意

14. **在我的大学学习中……**

**14.1** 老师经常鼓励我们提出不同于标准答案的观点。
**14.2** 课堂学习强调把知识用于解决真实问题。
**14.3** 课程学习重视合作、沟通与表达能力。
**14.4** 老师会鼓励我们独立思考，而不仅仅是记住知识点。
**14.5** 课程评价主要看是否记住了大量事实和标准答案。
**14.6** 我所在专业的学习更强调考试结果，而不是理解与应用。
**14.7** 在课堂上，尝试不同思路通常会受到鼓励。
**14.8** 犯错通常被视为学习和改进的一部分。

### E. 内在动机量表

**说明：**
以下题目想了解您为什么愿意持续投入大学学习。请根据您的真实情况选择。

**Response options for Items 15.1-15.8**
1 = 非常不符合
2 = 不太符合
3 = 一般
4 = 比较符合
5 = 非常符合

**15. 我之所以愿意投入大学学习，是因为……**

**15.1** 我享受发现新知识、新观点带来的乐趣。
**15.2** 理解复杂问题会让我感到满足。
**15.3** 解决学习中的难题会让我获得成就感。
**15.4** 学习本身对我来说是一件有趣的事。
**15.5** 我喜欢探索课程内容背后的原理和逻辑。
**15.6** 当我学会一个原本不懂的概念时，我会很开心。
**15.7** 即使没有外在奖励，我也愿意去学习感兴趣的内容。
**15.8** 持续学习能让我感受到自我成长。

### F. 学习投入量表

**说明：**
以下题目想了解您在日常学习中的投入状态。请根据您的实际体验选择最符合的一项。

**Response options for Items 16.1-16.9**
1 = 从不
2 = 很少
3 = 有时
4 = 经常
5 = 总是

**16. 在学习过程中……**

**16.1** 我感到自己精力充沛。
**16.2** 一想到开始一天的学习，我通常有动力行动起来。
**16.3** 我觉得自己所学的内容是有意义的。
**16.4** 我对自己的学习内容怀有热情。
**16.5** 学习时，我常常能保持较长时间的专注。
**16.6** 当我认真学习时，时间往往过得很快。
**16.7** 即使学习任务较多，我也愿意坚持完成。
**16.8** 我能够沉浸在当前的学习活动中。
**16.9** 学习会激发我的兴趣和灵感。

### G. 注意力检测题

**17. 为确保问卷质量，请在本题选择“比较符合”。**
1 = 非常不符合
2 = 不太符合
3 = 一般
4 = 比较符合
5 = 非常符合

### H. 补充问题

**18. 总体而言，您认为当前大学教育更接近以下哪种取向？**
1 = 更偏重知识记忆与考试成绩
2 = 两者大致平衡
3 = 更偏重能力发展与综合素养

**19. 您认为当前大学学习中，最能提升您学习积极性的因素是？**
(可多选)
1 = 课程内容有现实意义
2 = 老师鼓励独立思考
3 = 评价方式更加多元
4 = 学习内容有挑战性
5 = 同伴合作与讨论
6 = 未来就业或升学需要
7 = 个人兴趣
8 = 其他：____

# **Supplementary Material 3**

## Russian-Language Questionnaire Used for the Kazakhstan Sample

### A. Participant Information and Consent

Здравствуйте!

Мы проводим академическое исследование, посвященное учебному опыту студентов университетов. Цель исследования — изучить внутреннюю мотивацию, учебную вовлеченность и восприятие современных образовательных практик студентами в разных образовательных контекстах. В опросе могут принимать участие только студенты университетов в возрасте 18 лет и старше. Заполнение анкеты занимает примерно 6-8 минут.

Анкета является анонимной. Все данные будут использоваться только в научных целях. Мы не собираем ваше имя, номер студенческого билета или другую информацию, позволяющую установить личность. Участие в исследовании полностью добровольное. Вы можете прекратить заполнение анкеты в любой момент без каких-либо негативных последствий.

**Этическая информация**

Участие в исследовании является полностью добровольным.

Вы можете отказаться от участия или прекратить заполнение анкеты в любой момент.

Анкета заполняется анонимно.

Полученные данные будут использоваться только в научных целях и анализироваться в обобщенном виде.

Участие в исследовании не связано с существенными рисками.

Если какой-либо вопрос вызывает дискомфорт, вы можете пропустить его или завершить участие.

### Consent Item

**1. Прочитали ли вы информацию выше и добровольно согласны участвовать в данном исследовании?**
1 = Да, я согласен(на) участвовать
2 = Нет, я не согласен(на) участвовать

### B. Screening Items

**2. Являетесь ли вы в настоящее время студентом университета?**
(бакалавриат, магистратура или докторантура)
1 = Да
2 = Нет

**3. Вам исполнилось 18 лет?**
1 = Да
2 = Нет

**4. Где расположен ваш университет?**
1 = Шанхай, Китай
2 = Казахстан
3 = Другая страна / другой регион

### C. Demographic Information

**5. Ваш текущий статус обучения:**
1 = Студент бакалавриата
2 = Студент магистратуры
3 = Докторант / PhD студент

**6. Ваш пол:**
1 = Мужской
2 = Женский
3 = Другое
4 = Предпочитаю не отвечать

**7. Ваш возраст:**

**8. На каком курсе / уровне обучения вы сейчас находитесь?**
1 = 1 курс бакалавриата
2 = 2 курс бакалавриата
3 = 3 курс бакалавриата
4 = 4 курс бакалавриата
5 = 1 курс магистратуры
6 = 2 курс магистратуры и выше
7 = Докторантура

**9. К какой категории относится ваша специальность?**
1 = Гуманитарные и социальные науки
2 = Естественные науки и инженерия
3 = Медицина и науки о здоровье
4 = Педагогические науки
5 = Искусство и спорт
6 = Другое

**10. Как бы вы оценили экономическое положение вашей семьи по сравнению со средним уровнем в вашем регионе?**
1 = Значительно ниже среднего
2 = Немного ниже среднего
3 = Среднее
4 = Немного выше среднего
5 = Значительно выше среднего

**11. Каков наивысший уровень образования вашего отца?**
1 = Основное среднее и ниже
2 = Среднее / среднее специальное
3 = Колледж / техникум
4 = Высшее образование
5 = Магистратура и выше
6 = Не знаю / предпочитаю не отвечать

**12. Каков наивысший уровень образования вашей матери?**
1 = Основное среднее и ниже
2 = Среднее / среднее специальное
3 = Колледж / техникум
4 = Высшее образование
5 = Магистратура и выше
6 = Не знаю / предпочитаю не отвечать

**13. Как бы вы в целом оценили свою текущую академическую успеваемость?**
1 = Очень низкая
2 = Скорее низкая
3 = Средняя
4 = Скорее высокая
5 = Очень высокая

### D. Perceived Educational Transition Scale

**Instruction.**
Ниже приведены утверждения о вашем восприятии современного университетского образования. Пожалуйста, выберите вариант ответа, который наиболее точно отражает ваш опыт.

**Response options for Items 14.1-14.8**
1 = Полностью не согласен(на)
2 = Не согласен(на)
3 = Затрудняюсь ответить / нейтрально
4 = Согласен(на)
5 = Полностью согласен(на)

**14. В процессе моего обучения в университете…**

**14.1** Преподаватели часто поощряют нас высказывать мнения, отличающиеся от стандартных ответов.
**14.2** Обучение на занятиях подчеркивает применение знаний для решения реальных проблем.
**14.3** В учебном процессе большое внимание уделяется сотрудничеству, коммуникации и навыкам самовыражения.
**14.4** Преподаватели поощряют нас мыслить самостоятельно, а не просто запоминать учебный материал.
**14.5** Оценивание по предметам в основном зависит от того, насколько хорошо студент запомнил большое количество фактов и стандартных ответов.
**14.6** Обучение по моей специальности больше ориентировано на результаты экзаменов, чем на понимание и применение знаний.
**14.7** На занятиях обычно поощряется поиск разных способов решения задач.
**14.8** Ошибки обычно рассматриваются как часть процесса обучения и совершенствования.

### E. Intrinsic Motivation Scale

**Instruction.**
Следующие утверждения касаются того, почему вы готовы продолжать учиться в университете. Пожалуйста, ответьте в соответствии с вашей реальной ситуацией.

**Response options for Items 15.1-15.8**
1 = Совсем не про меня
2 = Скорее не про меня
3 = И да, и нет
4 = Скорее про меня
5 = Полностью про меня

**15. Я готов(а) вкладываться в обучение в университете, потому что…**

**15.1** Мне нравится удовольствие от открытия новых знаний и новых идей.
**15.2** Понимание сложных вопросов приносит мне удовлетворение.
**15.3** Решение трудных учебных задач дает мне чувство достижения.
**15.4** Сам процесс обучения для меня интересен.
**15.5** Мне нравится разбираться в принципах и логике, лежащих в основе учебного материала.
**15.6** Когда я понимаю концепцию, которую раньше не понимал(а), я испытываю радость.
**15.7** Даже без внешнего вознаграждения я готов(а) изучать то, что мне интересно.
**15.8** Постоянное обучение позволяет мне ощущать собственный рост.

### F. Learning Engagement Scale

**Instruction.**
Следующие утверждения касаются вашей вовлеченности в повседневное обучение. Пожалуйста, выберите вариант, который лучше всего отражает ваш реальный опыт.

**Response options for Items 16.1-16.9**
1 = Никогда
2 = Редко
3 = Иногда
4 = Часто
5 = Всегда

**16. В процессе учебы…**

**16.1** Я чувствую себя полным(ой) энергии.
**16.2** Когда я думаю о начале учебного дня, у меня обычно есть мотивация приступить к делу.
**16.3** Я считаю, что то, что я изучаю, имеет смысл и значение.
**16.4** Я испытываю энтузиазм по отношению к тому, что изучаю.
**16.5** Во время учебы мне часто удается сохранять сосредоточенность в течение длительного времени.
**16.6** Когда я серьезно учусь, время обычно проходит очень быстро.
**16.7** Даже если учебных заданий много, я готов(а) настойчиво доводить их до конца.
**16.8** Я способен(на) полностью погружаться в текущую учебную деятельность.
**16.9** Учеба пробуждает мой интерес и вдохновение.

### G. Attention Check Item

**17. Для контроля качества данных, пожалуйста, выберите вариант “Скорее про меня”.**
1 = Совсем не про меня
2 = Скорее не про меня
3 = И да, и нет
4 = Скорее про меня
5 = Полностью про меня

### H. Supplementary Questions

**18. В целом, какому типу, на ваш взгляд, ближе современное университетское образование?**
1 = Больше ориентировано на запоминание знаний и экзаменационные результаты
2 = Примерно сбалансировано
3 = Больше ориентировано на развитие компетенций и комплексных навыков

**19. Какие факторы, по вашему мнению, больше всего повышают вашу учебную мотивацию в университете?**
(Можно выбрать несколько вариантов)
1 = Практическая значимость содержания курса
2 = Поощрение самостоятельного мышления со стороны преподавателей
3 = Более разнообразные способы оценивания
4 = Сложность и интеллектуальный вызов учебного материала
5 = Совместная работа и обсуждение с однокурсниками
6 = Необходимость для будущей карьеры или продолжения обучения
7 = Личный интерес
8 = Другое: ____

## **Appendix B: Codebook and Scoring Rules**

### **A. Demographic Variables**

| Variable | Item | Coding |
| --- | --- | --- |
| region | Q4 | 1 = Shanghai, 2 = Kazakhstan, 3 = Other |
| study_level | Q5 | 1 = Undergraduate, 2 = Master’s, 3 = Doctoral |
| gender | Q6 | 1 = Male, 2 = Female, 3 = Other, 4 = Prefer not to say |
| age | Q7 | Numeric response |
| year_study | Q8 | 1-7 as listed in questionnaire |
| major_cat | Q9 | 1-6 as listed in questionnaire |
| family_econ | Q10 | 1-5, lower to higher |
| father_edu | Q11 | 1-6 as listed in questionnaire |
| mother_edu | Q12 | 1-6 as listed in questionnaire |
| acad_perf | Q13 | 1-5, lower to higher |

### **B. Scale Variables**

| Scale | Items | Coding | Reverse-coded items | Score computation |
| --- | --- | --- | --- | --- |
| Perceived Educational Transition | Q14.1-Q14.8 | 1-5 | Q14.5, Q14.6 | Mean of all items after reverse-coding |
| Intrinsic Motivation | Q15.1-Q15.8 | 1-5 | None | Mean of all items |
| Learning Engagement | Q16.1-Q16.9 | 1-5 | None | Mean of all items |

### **C. Attention Check**

| Variable | Item | Rule |
| --- | --- | --- |
| attention_check | Q17 | Response 4 = pass |

### **D. Supplementary Variables**

| Variable | Item | Coding |
| --- | --- | --- |
| edu_orientation | Q18 | 1 = memorization/exam oriented, 2 = balanced, 3 = competence oriented |
| mot_factor_1 to mot_factor_8 | Q19 | 0 = not selected, 1 = selected |

### **E. Scoring Note**

For reverse-coded items, responses were recoded as follows:
1 -> 5, 2 -> 4, 3 -> 3, 4 -> 2, 5 -> 1.
